# Multi‐Target Mechanisms of Whey Protein Against NAFLD: Integrating Bile Acid Metabolism, Gut Microbiota and Hepatic Inflammation

**DOI:** 10.1002/fsn3.71655

**Published:** 2026-03-16

**Authors:** Dongjin Xu, Biru Qiu, Xueyun Dong, Jiajun Tan, Yunhan Xie, Yuanyuan Wan, Chenghai Chu, Chunrun Miao, Asmaa Ali, Min Chen, Jiayuan He, Liang Wu, Jiayong Xie

**Affiliations:** ^1^ Department of Laboratory Medicine Dongtai Hospital of Traditional Chinese Medicine Yancheng Jiangsu China; ^2^ Department of Laboratory Medicine, School of Medicine Jiangsu University Zhenjiang China; ^3^ “Zhi Wei Bing” Focuses on Preemptive Healthcare in Traditional Chinese Medicine Dongtai Hospital of Traditional Chinese Medicine Yancheng Jiangsu China; ^4^ Gastrointestinal Department Dongtai Hospital of Traditional Chinese Medicine Yancheng Jiangsu China; ^5^ Department of Pulmonary Medicine Abbassia Chest Hospital, EMOH Cairo Egypt; ^6^ Public Experiment and Service Center Jiangsu University Zhenjiang China; ^7^ Health Testing Center Zhenjiang Center for Disease Control and Prevention Zhenjiang China; ^8^ Nephrology Department Xinghua People's Hospital Affiliated to Yangzhou University Taizhou China

**Keywords:** bile acids, gut microbiota, metabolomics, NAFLD, network pharmacology, short‐chain fatty acids, whey protein

## Abstract

This study elucidates the protective mechanisms of whey protein (WP) in treating high‐fat diet (HFD)‐induced nonalcoholic fatty liver disease (NAFLD) in mice, emphasizing its role in bile acid regulation, intestinal flora homeostasis, and inflammatory suppression. ICR mice were subjected to a 12‐week HFD to establish NAFLD, followed by WP intervention (200 g/kg). Comprehensive analyses included histopathological assessment (HE staining), serum biomarkers, hepatic gene expression (qPCR), gut microbial profiling (16S rRNA sequencing), quantitative bile acid and short‐chain fatty acid (SCFA) analysis, and serum metabolomics. Core targets were predicted via network pharmacology and validated through molecular docking. WP administration markedly alleviated NAFLD progression by targeting multiple pathways: (1) It suppressed hepatic lipid deposition and inflammatory injury, downregulating NLRP3, NF‐κB, and TNF‐α (*p* < 0.05) while enhancing Nrf2/HO‐1‐mediated antioxidant defenses; (2) Network pharmacology prioritized IL‐1β, STAT3, and MMP9 as pivotal targets, with β‐lactoglobulin exhibiting high binding potentials (STAT3: −1.42 kcal/mol); (3) WP restored gut microbial balance, enriching beneficial taxa (e.g., *Lactobacillus*) and fecal SCFAs; (4) It reprogrammed bile acid metabolism, elevating cholesterol‐cleaving enzymes (CYP7A1/CYP27A1) but inhibiting FXR/SHP (*p* < 0.05), alongside increased hepatoprotective bile acids (TDCA/TUDCA). Metabolomics identified WP‐induced anti‐inflammatory mediators (e.g., eicosapentaenoic acid) and perturbations in arginine and unsaturated fatty acid pathways, synergistically attenuating steatosis and fibrosis. WP counters NAFLD via a tripartite mechanism: gut microbiome‐directed SCFA synthesis, bile acid‐driven cholesterol disposal, and dual modulation of inflammation (NLRP3/NF‐κB) and oxidative stress (Nrf2/HO‐1). These insights position WP as a promising dietary strategy targeting the gut‐liver axis.

## Graphical abstract

Whey protein (WP) alleviates high‐fat diet (HFD)‐induced nonalcoholic fatty liver disease (NAFLD) through a tripartite mechanism targeting the gut‐liver axis. (1) Gut Microbiota Modulation: WP restores intestinal flora homeostasis by enriching beneficial taxa (e.g., *Lactobacillus*) and increasing the production of short‐chain fatty acids (SCFAs). (2) Bile Acid Reprogramming: WP promotes cholesterol catabolism by upregulating CYP7A1/CYP27A1 and downregulating FXR/SHP, leading to elevated levels of hepatoprotective secondary bile acids (TDCA/TUDCA). (3) Hepatic Inflammation and Oxidative Stress: WP's core bioactive component, β‐lactoglobulin, interacts with key targets (e.g., STAT3, MMP9). Systemically, WP suppresses the NLRP3/NF‐κB inflammatory cascade and activates the Nrf2/HO‐1 antioxidant defenses. Collectively, these synergistic pathways attenuate hepatic steatosis, inflammatory injury, and fibrogenesis, effectively counteracting NAFLD progression.

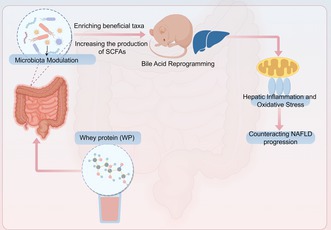

## 1 Introduction

Nonalcoholic fatty liver disease (NAFLD), characterized by excessive hepatic lipid accumulation in the absence of excessive alcohol consumption, has emerged as a leading cause of chronic liver disease globally, with a prevalence exceeding 25% in adults (Geng et al. 2021; Mantovani et al. 2022; Sanyal et al. 2021). Its pathological progression ranges from simple steatosis to nonalcoholic steatohepatitis (NASH), fibrosis, and cirrhosis, with increasing risks of hepatocellular carcinoma (Burra et al. 2021; Grgurevic et al. 2021; Tacke and Weiskirchen 2021). While the “multiple‐hit” hypothesis suggests that NAFLD develops through complex interactions among genetic predisposition, insulin resistance, oxidative stress, and gut dysbiosis, effective pharmacological interventions remain limited (Alabdulaali et al. 2023; Li and Zhao 2021; Rodriguez‐Ramiro 2023). Therapeutic strategies targeting metabolic disturbances, gut‐liver crosstalk, and inflammation are thus urgently needed.

Whey protein (WP), a nutrient‐rich milk derivative, has shown promise in ameliorating metabolic disorders due to its high bioavailability, anti‐inflammatory peptides (e.g., lactoferrin), and cysteine content that supports glutathione synthesis (Karimidastjerd and Gulsunoglu‐Konuskan 2023; Sah et al. 2018; Yiğit et al. 2023). Clinical trials indicate that WP supplementation reduces hepatic fat content and inflammatory markers in NAFLD patients, but its mechanisms—particularly its interplay with gut microbiota and bile acid metabolism—are incompletely understood (Giglio et al. 2023; Kim et al. 2023; Mizubuti et al. 2021). Emerging evidence suggests that WP may modulate the “bile acid–gut microbiota–liver axis,” a critical pathway governing cholesterol catabolism, microbial composition, and hepatic inflammation (Gallo et al. 2024; Ji et al. 2023; Li et al. 2025). For instance, WP‐derived β‐lactoglobulin, a lipocalin‐family protein, may bind signaling molecules like STAT3 or RAGE to suppress NLRP3 inflammasome activation, yet direct experimental validation is lacking (Almohawes et al. 2025; Quintieri et al. 2025).

Here, we employed a high‐fat diet (HFD)‐induced NAFLD mouse model to systematically investigate WP's protective effects through multi‐omics approaches. Our findings provide mechanistic insights into WP's therapeutic potential, positioning it as a dietary intervention for NAFLD through gut‐liver axis regulation.

## 2 Material and Methods

### 2.1 Network Pharmacology and Molecular Docking Analysis of WP'S Mechanisms in NAFLD Treatment

To systematically elucidate the multi‐component, multi‐target, and multi‐pathway mechanisms of whey protein (WP) against NAFLD, an integrated network pharmacology and molecular docking approach was employed. The primary constituents of WP (α‐lactalbumin, β‐lactoglobulin, lactoferrin, etc.) were first retrieved from the UniProt database (https://www.uniprot.org/). Potential therapeutic targets were predicted using PharmMapper and SwissTargetPrediction platforms, followed by deduplication and standardization to establish a WP target dataset. Disease‐related targets for NAFLD were obtained by searching DisGeNET (v7.0), GeneCards (v5.16), and OMIM databases with the keyword “non‐alcoholic fatty liver disease,” and consolidated into a deduplicated NAFLD target dataset. The intersection of WP and NAFLD targets was identified as key targets and imported into the STRING database (v12.0) to construct a protein–protein interaction (PPI) network with a confidence threshold > 0.7. Core targets were screened using Cytoscape 3.9.1 based on Degree and Betweenness centrality metrics.

An integrative, multi‐level network was constructed using Cytoscape to elucidate interactions among WP‐derived bioactive compounds, prioritized targets, and NAFLD‐associated pathways. Topological metrics—including average shortest path length and clustering coefficient—were computed to pinpoint central regulatory hubs. Functional enrichment was assessed via Gene Ontology (GO; covering biological processes, molecular functions, and cellular components) and KEGG pathway mapping in DAVID 6.8 (*p* < 0.05, FDR < 0.01). Significantly perturbed pathways were graphically represented using the R package clusterProfiler.

For molecular docking validation, the top five targets by node centrality (Degree metric, e.g., PPARG, AKT1, IL6) were prioritized. High‐quality crystallographic structures (resolution ≤ 2.5 Å) were retrieved from the RCSB PDB (https://www.rcsb.org/). Proteins were prepared through solvation shell removal and protonation state optimization, while ligands underwent energy minimization (MMFF94 force field). Docking simulations were executed in AutoDock Vina 1.2.0 with a 20 Å^3^ grid box centered on the active site (exhaustiveness: 8). Complexes exhibiting favorable binding thermodynamics (ΔG < 0 kcal/mol) were retained, followed by detailed interaction profiling (hydrogen bonding, hydrophobic interfaces, π‐effects) via PyMOL 2.5.

### 2.2 Experimental Animals and Grouping

Male ICR mice (6‐week‐old, 22–25 g) were housed under specific pathogen‐free (SPF) conditions (22°C ± 1°C, 50% ± 5% humidity, 12 h light/dark cycle) to establish a nonalcoholic fatty liver disease (NAFLD) model using a 60% high‐fat diet (HFD, Data S1) according to the protocol by Sun et al. (2023), with ethical approval obtained from Jiangsu University Animal Ethics Committee (protocol code UJS‐IACUCAP‐2023032011 and date of approval: January 2023). The mice were randomly divided into three groups receiving: (1) standard chow (NC group), (2) HFD (NAFLD group), or (3) whey protein‐supplemented HFD (WP group), where casein was replaced with 200 g/kg whey protein isolate (Nutrasumma Inc., City of Industry, CA, USA; processed by Beijing Fubo Biotechnology Co., Beijing, China). The nutritional composition of the feed is provided in Supporting Information.

After 12 weeks of dietary intervention, mice were euthanized by intraperitoneal injection of urethane (700 mg/kg, Sigma‐Aldrich, St. Louis, MO, USA). Fecal samples were collected for gut microbiota analysis, serum was obtained for biochemical characterization and untargeted metabolomics, and liver tissues were harvested for histological examination by hematoxylin–eosin (HE) staining. This experimental design enabled comprehensive evaluation of WP's potential therapeutic effects on NAFLD development through multi‐omics approaches.

### 2.3 Serum Biochemical and Oxidative Stress Profile Analysis

Hepatic function and metabolic parameters were evaluated by quantifying serum markers, including hepatic transaminases (alanine aminotransferase [ALT, A020‐1] and aspartate aminotransferase [AST, A021‐1]), lipid profile markers (triglycerides [TG, A110‐1‐1], total cholesterol [TC, A111‐1], high‐density lipoprotein cholesterol [HDL‐C, A112‐1], and low‐density lipoprotein cholesterol [LDL‐C, A113‐1]), as well as oxidative stress indicators (superoxide dismutase [SOD, A001‐1] activity and malondialdehyde [MDA, A003‐1]). Quantification was performed using standardized enzymatic assay kits (Nanjing Jiancheng Bioengineering Institute, Nanjing, China) in strict accordance with the manufacturer's instructions.

### 2.4 Hepatic RNA Isolation and Quantitative Gene Expression Analysis

Hepatic RNA was extracted from liver specimens using the RNApure Total RNA Kit (Vazyme Biotech, China) following standard protocols. Reverse transcription reactions were carried out on 1 μg aliquots of purified RNA employing HiScript III RT SuperMix (Vazyme, China), utilizing optimized conditions of 42°C for 15 min followed by a 5‐s enzyme inactivation step at 85°C.

For quantitative PCR analyses, reaction mixtures (20 μL total volume) were prepared containing ChamQ Universal SYBR qPCR Master Mix (Vazyme, China), forward and reverse primers (0.4 μL each at 10 μM concentration), cDNA template (2 μL), and molecular grade water. Thermal cycling was performed on a CFX96 instrument with the following parameters: initial denaturation (95°C, 30 s), 40 cycles of two‐step amplification (95°C, 10 s; 60°C, 30 s), concluded by melt curve generation (65°C–95°C range with 0.5°C increments) to ensure reaction specificity.

Targeted gene expression analysis focused on inflammation‐related factors, oxidative stress markers, and bile acid metabolism enzymes using custom‐designed primers synthesized by GENEWIZ. Comprehensive primer information including sequences and predicted amplicon sizes is provided in Table S1.

### 2.5 16S rRNA Sequencing and Microbiota Analysis

Colonic microbiota composition was assessed by high‐throughput sequencing of the bacterial 16S rRNA gene (ekemo, China). Briefly, 100 mg colonic contents from each mouse (*n* = 6 per group) were homogenized in 1 mL phosphate‐buffered saline (PBS), followed by centrifugation at 800 *g* for 5 min to pellet particulate matter. The resulting supernatant was subjected to microbial DNA extraction.

The hypervariable V3–V4 region was amplified via PCR using universal primers 341F (5′‐CCTACGGGNGGCWGCAG‐3′) and 806R (5′‐GGACTACHVGGGTWTCTAAT‐3′). PCR products were purified and paired‐end sequenced (2 × 250 bp) on an Illumina NovaSeq 6000 platform (Illumina, San Diego, CA, USA). Raw sequence data were quality filtered (Q‐score ≥ 20; truncation length = 240 bp), denoised using DADA2, and chimeric sequences were removed via the QIIME2 pipeline (v2023.5). High‐quality amplicon sequence variants (ASVs) were clustered at 100% similarity and taxonomically classified against the SILVA 138 database.

Microbial community diversity was evaluated using α‐diversity (Shannon index) and β‐diversity (principal component analysis, PCA). Differentially abundant bacterial taxa were identified via linear discriminant analysis effect size (LEfSe) with a threshold of LDA score > 3.0 (*p* < 0.05).

### 2.6 Untargeted Metabolomics and Targeted LC–MS/MS Profiling

Blood specimens were collected from the retro‐orbital sinus after a 12‐h fast and subsequently centrifuged (3000 *g*, 10 min, 4°C) to obtain serum. Metabolite extraction was performed by adding 300 μL of ice‐cold methanol:acetonitrile (1:1) to 100 μL serum aliquots. Following brief vortexing (30 s) and cold exposure (−20°C, 1 h), protein precipitation was achieved by high‐speed centrifugation (14,000 *g*, 15 min, 4°C). The clarified supernatants were freeze‐dried and redissolved in 100 μL of 50% acetonitrile for subsequent LC–MS analysis (ekemo Tech, China).

LC–MS raw data were processed using Compound Discoverer 3.3 (Thermo Scientific, USA) with a three‐step workflow: (1) initial peak detection from full‐scan spectra, (2) chromatographic alignment across samples, and (3) compound identification through comparison with reference databases (HMDB, KEGG, LipidMaps).

Multivariate pattern recognition was implemented in MetaboAnalyst 5.0, incorporating both unsupervised (PCA) and supervised (OPLS‐DA) approaches. Statistically relevant metabolites (VIP > 1.0, *p* < 0.05 by two‐tailed *t*‐test) were further examined through metabolic pathway mapping.

Targeted quantification of circulating bile acids and microbial fermentation products (acetate/propionate/butyrate) was conducted by LC–MS/MS at the Zhenjiang CDC (China), with detailed methodological parameters available in the Supporting Information.

### 2.7 Histopathological Examination (H&E Staining)

Liver tissue specimens obtained from NAFLD murine models were subjected to standard histopathological processing. Following 24‐h fixation in 4% paraformaldehyde at 4°C, samples underwent sequential dehydration using increasing ethanol concentrations (70% to absolute ethanol), subsequent xylene clearing, and paraffin embedding. Tissue sections of 4 μm thickness were prepared using a rotary microtome (Leica, Germany) and mounted on pre‐cleaned glass slides.

Histological assessment was performed following hematoxylin and eosin (H&E) staining conducted by certified pathologists at our institutional pathology department. Morphological evaluation was carried out using bright‐field microscopy (Nikon Eclipse, Japan), with particular attention to characteristic NAFLD pathological features including steatosis, lobular inflammation, and hepatocellular ballooning.

### 2.8 Statistical Analysis

Statistical analysis of routine biochemical and molecular data was carried out using SPSS 26.0 (IBM Corp., Armonk, NY, USA), with continuous numerical data expressed as means ± SD. Group differences were assessed through one‐way analysis of variance (ANOVA), followed by Tukey's HSD post hoc tests when significant main effects were detected (*p* < 0.05, two‐tailed). For the multi‐omics datasets, specific statistical pipelines were applied: (1) 16S rRNA microbiota analysis: Microbial community diversity was evaluated using α‐diversity indices and β‐diversity (PCoA), while differentially abundant bacterial taxa were identified via linear discriminant analysis effect size (LEfSe) with a threshold of LDA score > 3.0 (*p* < 0.05). (2) Metabolomics analysis: Multivariate pattern recognition, including unsupervised PCA and supervised OPLS‐DA, was implemented in MetaboAnalyst 5.0. Statistically relevant differential metabolites were screened based on a variable importance in projection (VIP) score > 1.0 and *p* < 0.05 by a two‐tailed Student's *t*‐test. (3) Network pharmacology: Functional enrichment analyses (GO and KEGG) were evaluated using the DAVID 6.8 database with strict significance thresholds set at *p* < 0.05 and False Discovery Rate (FDR) < 0.01.

## 3 Results

### 3.1 Body Weight and Hepatic Morphological Changes

**FIGURE 1:**
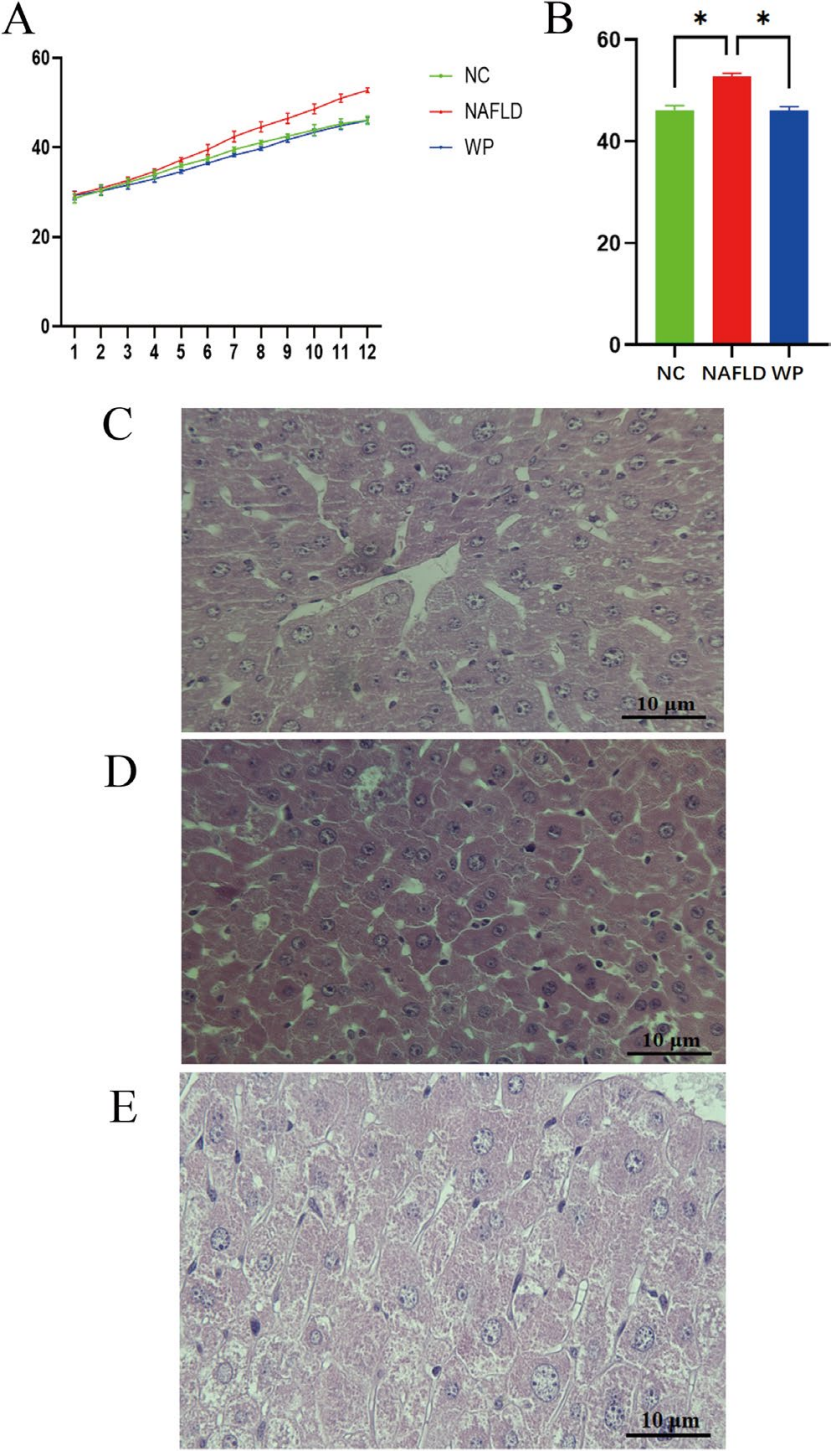
Mouse body weight and liver HE staining. (A) Body weight during the experimental period. (B) Body weight at the end of the experiment. (C) Liver HE staining in the NC group. (D) Liver HE staining in the NAFLD group. (E) Liver HE staining in the WP group.

Beginning in Week 7, body weight in the NAFLD group became notably reduced relative to both control (NC) and WP‐treated animals (*p* < 0.05). By study completion, body weights in the NC and WP groups were statistically equivalent, yet remained elevated compared to the NAFLD cohort (*p* < 0.05) (Figure 1A,B).

H&E‐stained liver sections revealed marked intergroup variation in tissue histology. Control animals (NC group) maintained normal lobular organization, with uniform hepatocyte morphology, dense cytoplasmic staining, and absent lipidosis or inflammation (Figure 1C). Hepatic tissue from NAFLD mice displayed hallmarks of disease progression—numerous heterogeneous lipid vacuoles, hepatocyte ballooning with cytoplasmic clearing, and morphological evidence of cellular damage (Figure 1D). Notably, WP administration substantially reversed these pathological alterations, showing only localized microvesicular steatosis, minimal hepatocyte swelling, and largely preserved liver microstructure (Figure 1E). These results collectively indicate WP‐mediated preservation of hepatic integrity during NAFLD development.

### 3.2 Network Pharmacology and Molecular Docking Analysis

**FIGURE 2:**
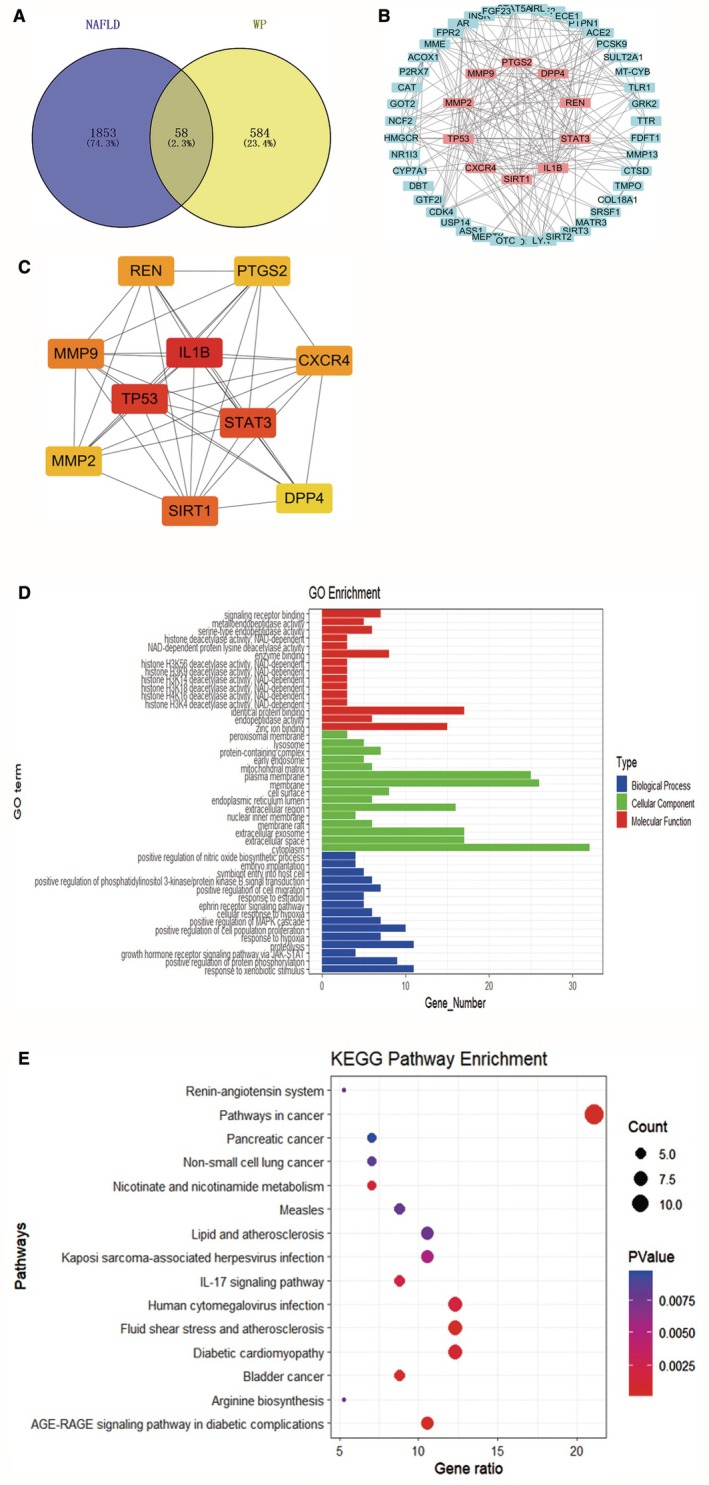
Network pharmacology results of WP in NAFLD treatment. (A) Venn diagram illustrating the overlapping targets between NAFLD‐related genes and WP potential therapeutic targets. (B) Protein–protein interaction (PPI) network of the overlapping targets. (C) Key hub targets screened from the PPI network. (D, E) Functional enrichment analysis of WP mechanisms in NAFLD treatment: (D) Gene ontology (GO) terms and (E) KEGG pathways.

Our computational screening approach pinpointed 642 potential whey protein (WP) targets using SwissTargetPrediction and PharmMapper, while disease databases (GeneCards, OMIM, TTD) yielded 1911 NAFLD‐related genes. Cross‐referencing identified 58 shared candidates likely mediating WP's anti‐NAFLD effects (Figure 2A). Network analysis of protein interactions (STRING/Cytoscape) produced a 53‐node, 223‐edge network highlighting key hubs—IL‐1β, TP53, STAT3, SIRT1, and MMP9 (Figure 2B,C)—implicating their centrality in WP's mode of action.

Functional clustering (DAVID) linked these targets to lipid homeostasis and inflammation modulation, with mitochondrial/exosomal localization and molecular roles spanning redox regulation and PPARγ activation (Figure 2D). Pathway mapping (KEGG) revealed 52 enriched routes, including IL‐17/AGE‐RAGE signaling, dyslipidemia‐related pathways, and fibrotic cascades (Figure 2E), suggesting WP's multifaceted impacts on metabolic inflammation and tissue remodeling.

**FIGURE 3:**
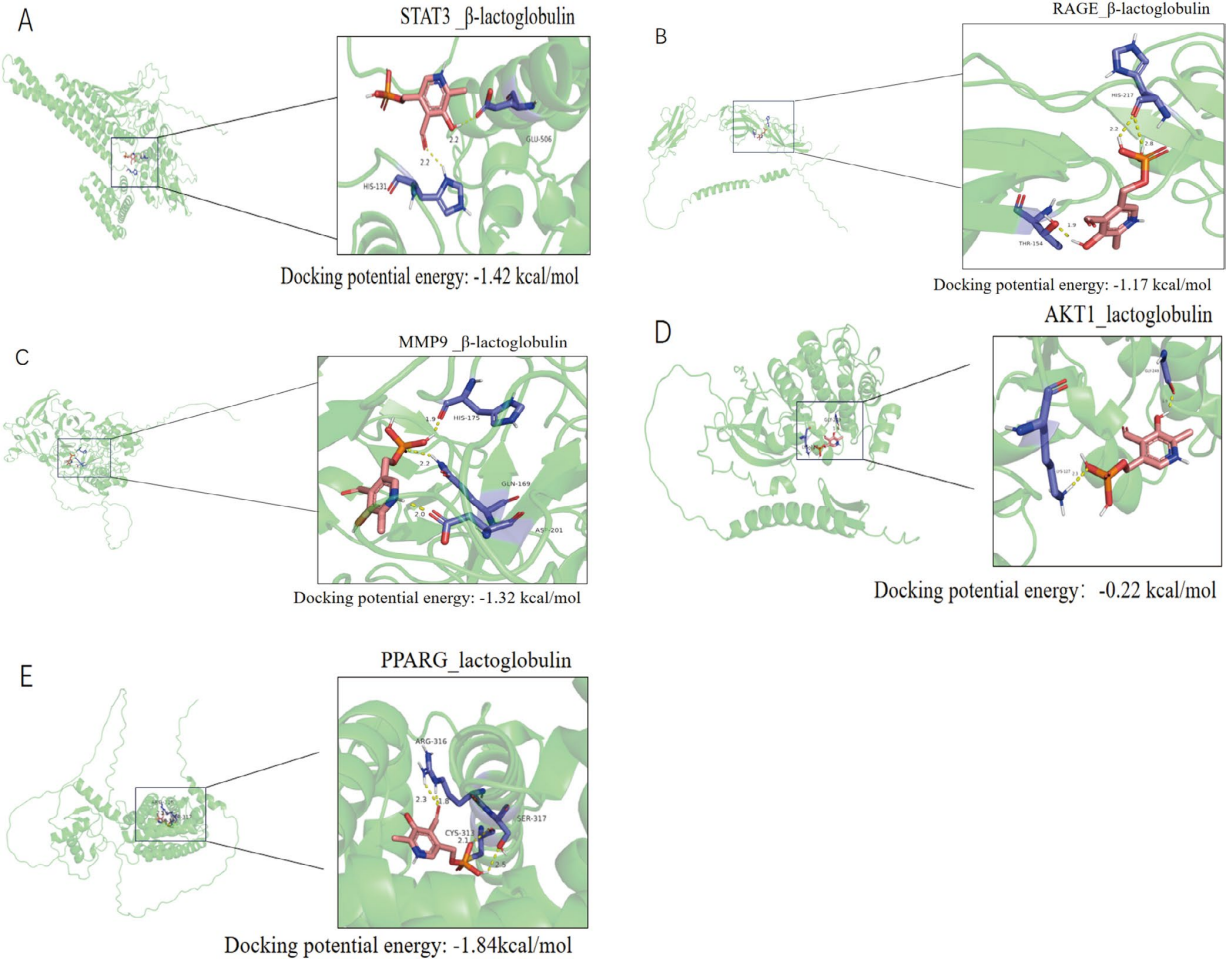
Molecular docking results of β‐lactoglobulin (a key component in WP) with hub targets. Detailed binding modes and docking potential energies are shown for (A) STAT3 (−1.42 kcal/mol), (B) RAGE (−1.17 kcal/mol), (C) MMP9 (−1.32 kcal/mol), (D) AKT1 (−0.22 kcal/mol), and (E) PPARG (−1.84 kcal/mol).

We next examined β‐lactoglobulin, a lipocalin‐family WP constituent mediating hydrophobic ligand transport. Docking simulations revealed its binding affinities to pivotal NAFLD targets, with the most robust interactions occurring at PPARG (Cys313/Arg316/Ser317, −1.84 kcal/mol; Figure 3E), STAT3 (Glu506/His330, −1.42 kcal/mol; Figure 3A), MMP9 (His175/Glu169/Asp201, −1.32 kcal/mol; Figure 3C), and RAGE (His217/Thr154, −1.17 kcal/mol; Figure 3B), while binding to AKT1 was comparatively weaker (−0.22 kcal/mol; Figure 3D). These structural insights mechanistically anchor WP's bioactivity to critical pathways.

### 3.3 Analysis of Serum Biochemical Parameters and Hepatic Inflammatory Factors in Mice

**FIGURE 4:**
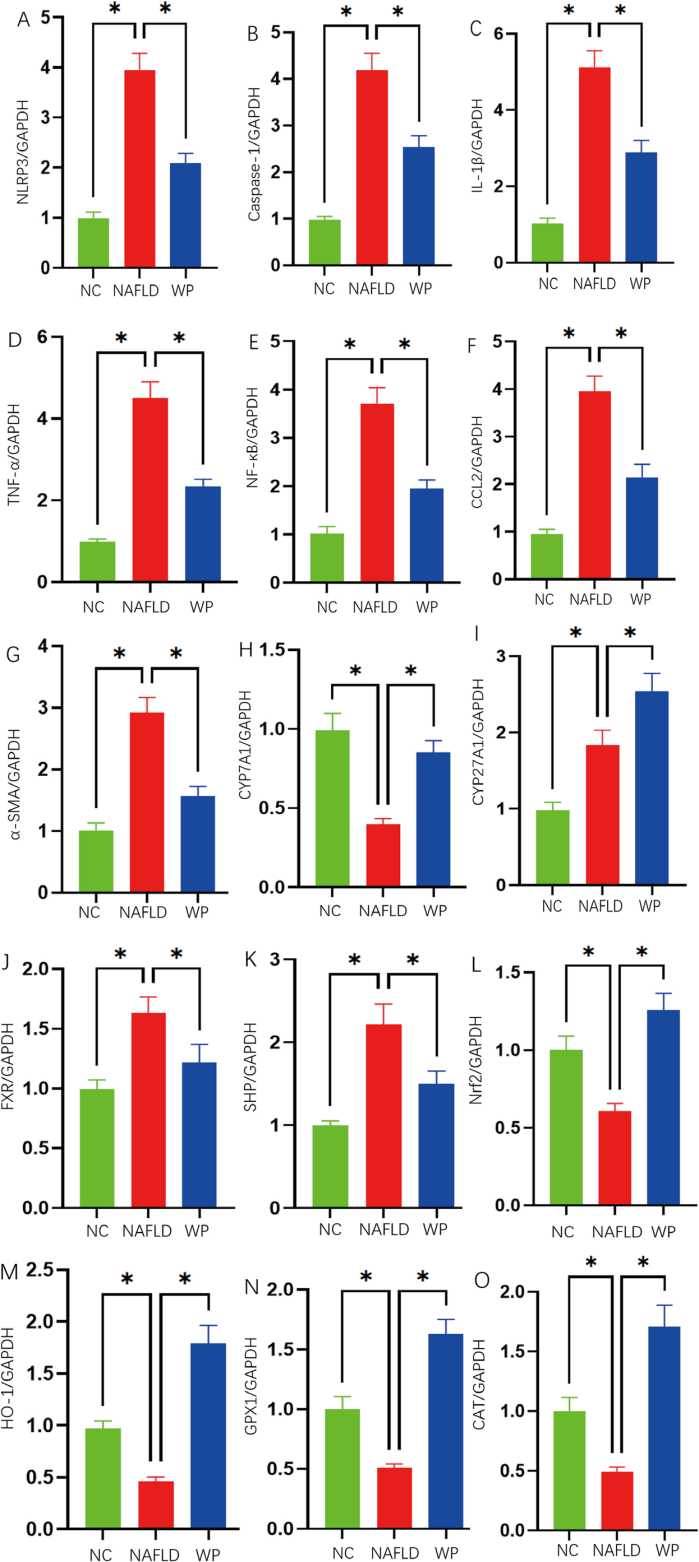
Quantitative real‐time PCR (qPCR) analysis of hepatic gene expression in mice. The relative mRNA expression levels of key genes were evaluated and normalized to GAPDH. The panels display specific genes categorized into four main functional pathways: (A–E) inflammatory factors, including (A) NLRP3, (B) Caspase‐1, (C) IL‐1β, (D) TNF‐α, and (E) NF‐κB; (F, G) fibrosis and macrophage infiltration‐related factors, including (F) CCL2 and (G) α‐SMA; (H–K) bile acid metabolism‐related factors, including the synthetic enzymes (H) CYP7A1 and (I) CYP27A1, as well as the receptors/regulators (J) FXR and (K) SHP; and (L–O) oxidative stress‐related and antioxidant defense genes, including (L) Nrf2, (M) HO‐1, (N) GPX1, and (O) CAT. Data are presented as means ± SD (*n* = 6 per group). * indicates statistical significance between the connected groups (*p* < 0.05).

Gene expression profiling revealed that WP administration led to significant downregulation of hepatic inflammatory markers NLRP3, Caspase‐1, IL‐1β, TNF‐α, NF‐κB, CCL2, and α‐SMA (*p* < 0.05 versus NAFLD; Figure 4A–G). The treatment simultaneously altered bile acid metabolism by enhancing CYP7A1 and CYP27A1 transcription while suppressing FXR and SHP expression (*p* < 0.05; Figure 4H–K). Transcript levels of antioxidant regulators Nrf2, HO‐1, GPX1, and CAT showed pronounced elevation in WP‐treated animals (*p* < 0.05; Figure 4L–O).

**FIGURE 5:**
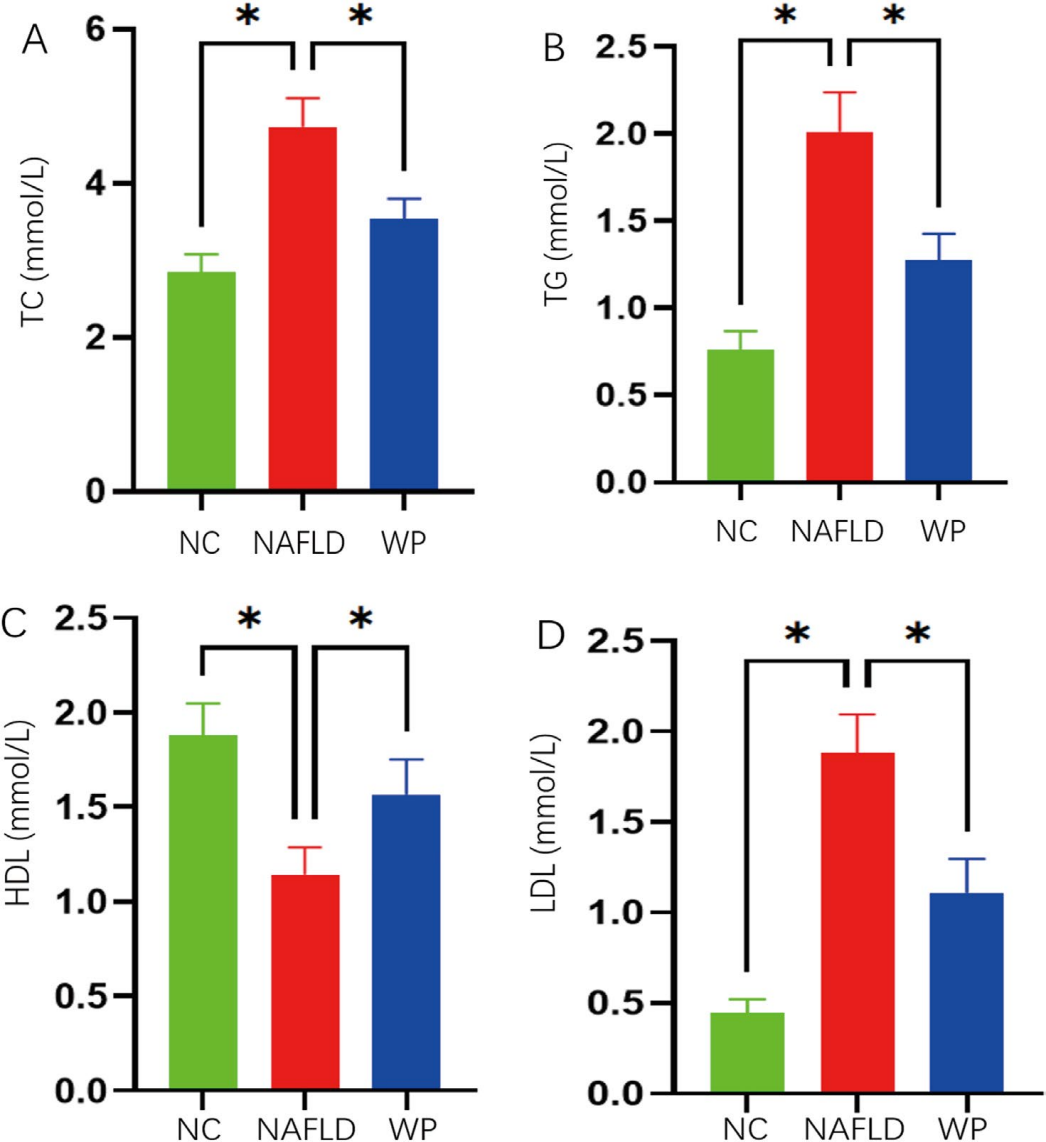
Serum lipid profiles in mice, including: (A) TC, (B) TG, (C) HDL, and (D) LDL.

Circulating lipid measurements demonstrated WP's beneficial metabolic effects, with substantial reductions in TC, TG, and LDL‐C coupled with increased HDL‐C concentrations (*p* < 0.05; Figure 5). These observations provide compelling evidence that WP supplementation attenuates liver inflammation while improving oxidative stress responses, bile acid homeostasis, and blood lipid profiles in the NAFLD model.

### 3.4 WP Modulates Gut Microbiota in NAFLD Mice

**FIGURE 6:**
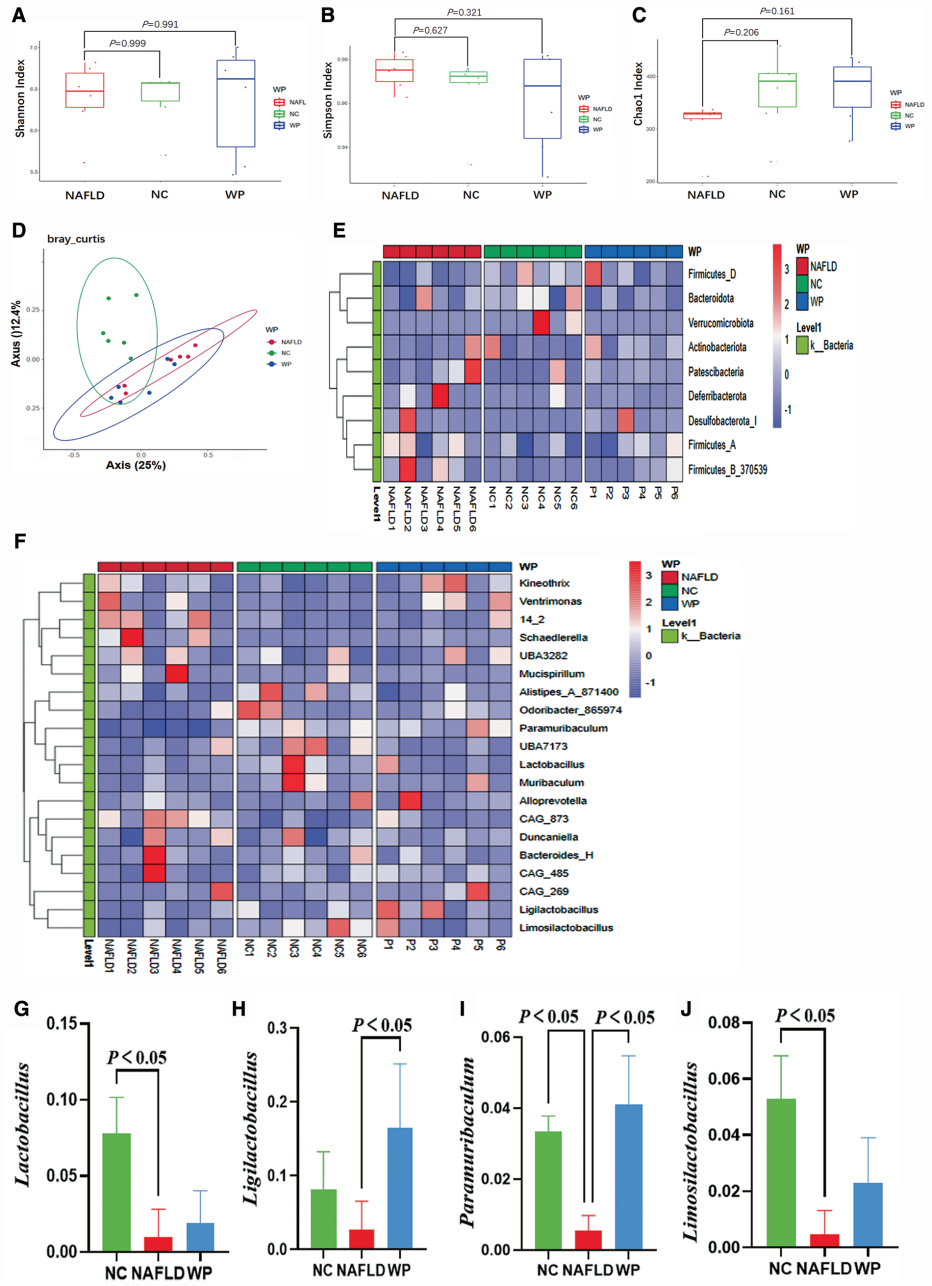
Gut microbiota analysis in mice via 16S rRNA sequencing. (A–C) α‐Diversity indices: Shannon (A), Simpson (B), and Chao1 (C). β‐Diversity analysis revealed significant separation of microbial communities among the NC, NAFLD, and WP groups (D). (E, F) Heatmaps of relative abundance at the phylum (E) and genus (F) levels. (G–J) Significantly altered genera: *Lactobacillus* (G), *Ligilactobacillus* (H), *Paramuribaculum* (I), and *Limosilactobacillus* (J) (*: *P* < 0.05).

To investigate WP impact on gut microbial ecology in NAFLD mice, we performed Illumina HiSeq‐based community profiling of bacterial OTUs. While analysis of α‐diversity metrics indicated no statistically significant differences in microbial richness and evenness between the NAFLD group and the NC or WP groups—specifically for the Shannon index (NAFLD vs. NC, *p* = 0.999; NAFLD vs. WP, *p* = 0.991), Simpson index (NAFLD vs. NC, *p* = 0.627; NAFLD vs. WP, *p* = 0.321), and Chao1 index (NAFLD vs. NC, *p* = 0.206; NAFLD vs. WP, *p* = 0.161) (Figure 6A–C)—β‐diversity patterns (PCoA) exhibited clear segregation. The NC and NAFLD cohorts clustered distinctly, whereas WP‐treated mice formed an intermediate group with unique compositional features (Figure 6D), implying WP‐mediated microbiota modulation.

Hierarchical clustering of phylum‐ and genus‐level abundances revealed pronounced intergroup variations (Figure 6E,F). Quantitative analysis identified NAFLD‐associated dysbiosis characterized by *Mucispirillum* enrichment alongside depletion of *Lactobacillus*, *Ligilactobacillus*, *Paramuribaculum*, and *Limosilactobacillus* relative to healthy controls (*p* < 0.05). WP treatment counteracted these imbalances, simultaneously suppressing mucispiral bacteria and restoring health‐promoting taxa (*p* < 0.05) (Figure 6G–J), demonstrating its ability to recalibrate the NAFLD‐disrupted microbiome.

### 3.5 Serum Bile Acid Profiles and Fecal SCFAs Concentrations in Mice

**FIGURE 7:**
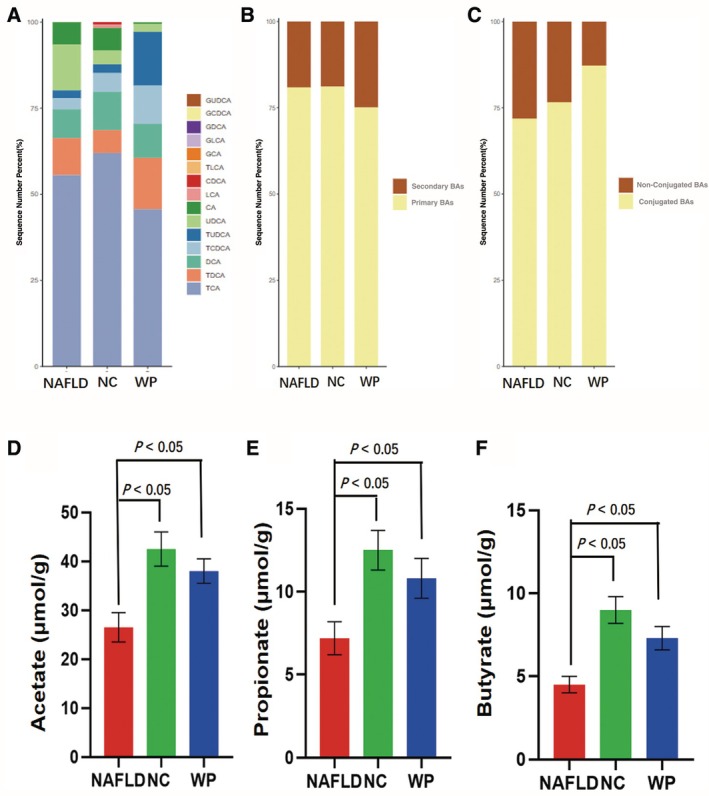
Serum bile acid profiles and colonic SCFAs concentrations in mice. (A) Total bile acid profile; (B) primary and secondary bile acids; (C) conjugated and unconjugated bile acids; (D) acetate; (E) propionate; (F) butyrate.

Comprehensive serum metabolomics profiling using UPLC–MS/MS unveiled distinct bile acid signatures across treatment groups (Figure 7A). WP administration substantially altered bile acid homeostasis, with treated animals displaying selective reductions in primary and conjugated bile acid pools compared to both healthy and NAFLD counterparts (Figure 7B,C). Specific analysis identified a characteristic WP‐associated pattern featuring enhanced TDCA and TUDCA levels, coupled with diminished UDCA and CA quantities versus NAFLD mice (*p* < 0.05).

Parallel examination of gut microbial metabolites demonstrated that HFD feeding significantly depleted fecal SCFA production compared to normal chow controls (*p* < 0.05). WP consumption reversed this metabolic perturbation, robustly normalizing the output of all major SCFAs in NAFLD‐affected animals (*p* < 0.05) (Figure 7D–F). These complementary shifts in circulating bile acid patterns and bacterial fermentation products highlight WP's integrative action in rewiring host‐microbial metabolic crosstalk during NAFLD progression.

### 3.6 WP Ameliorates Serum Metabolomic Disorders in NAFLD Mice

**FIGURE 8:**
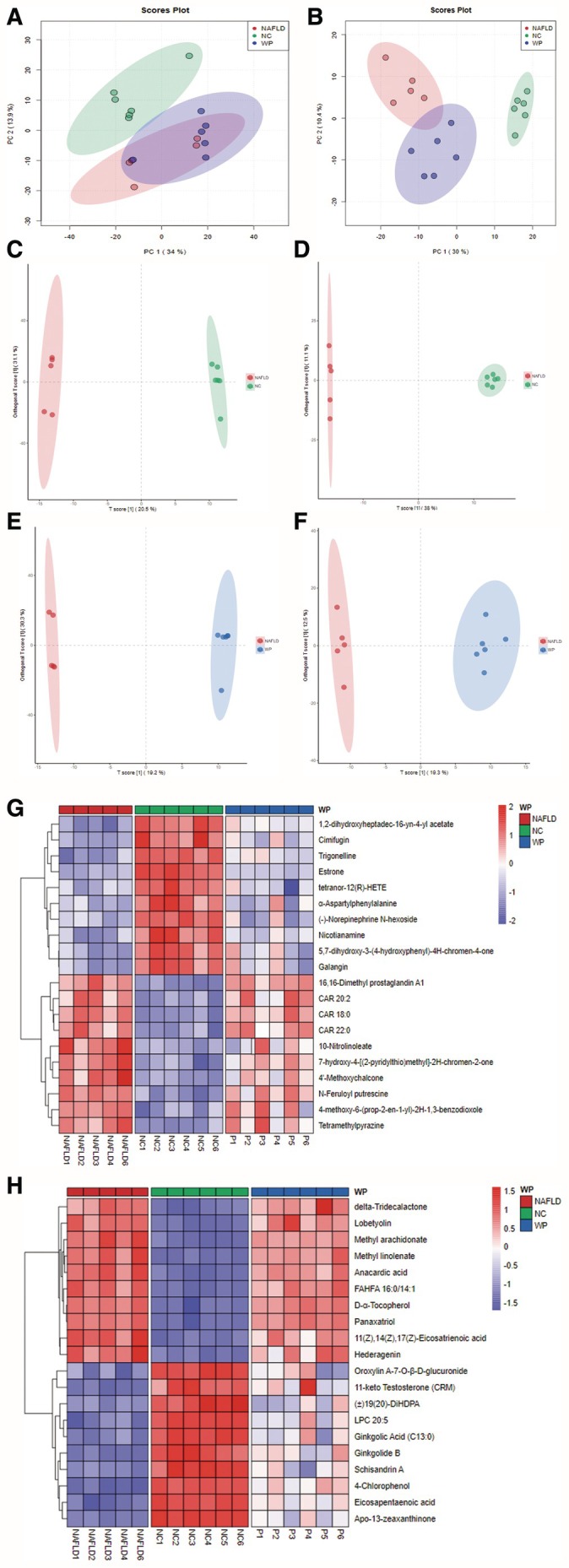
Serum untargeted metabolomics analysis in mice. (A, B) PCA score plots; (C–F) OPLS‐DA score plots; (G, H) Cluster heatmaps of differentially abundant metabolites.

Multivariate statistical analysis of the serum metabolome by PCA demonstrated clear separation among NC, NAFLD, and WP treatment cohorts in both ionization modes (Figure 8A,B). Intriguingly, the WP‐treated mice showed greater metabolic similarity to healthy controls than NAFLD mice, suggesting WP's ability to partially normalize the disease‐associated metabolomic signature. These findings were substantiated by OPLS‐DA modeling (Figure 8C–F), which identified significantly altered metabolites (VIP > 1, *p* < 0.05; Tables S2, S3; Figure 8G,H).

Hierarchical clustering of discriminant metabolites revealed WP's capacity to upregulate key beneficial compounds. In negative ionization mode, WP boosted levels of EPA, LPC 20:5, DiHDPA isomers, and 11‐ketotestosterone—metabolites linked to inflammation resolution and oxidative defense (*p* < 0.05). Positive mode analysis similarly showed WP‐induced increases in estrone, tetranor‐HETE derivatives, aspartylphenylalanine, and nicotianamine, bioactive molecules involved in lipid homeostasis and metabolic regulation (*p* < 0.05). Collectively, these results demonstrate WP's systemic metabolic remodeling effects that counteract NAFLD pathophysiology.

### 3.7 Serum Metabolic Pathway Enrichment in Mice

**FIGURE 9:**
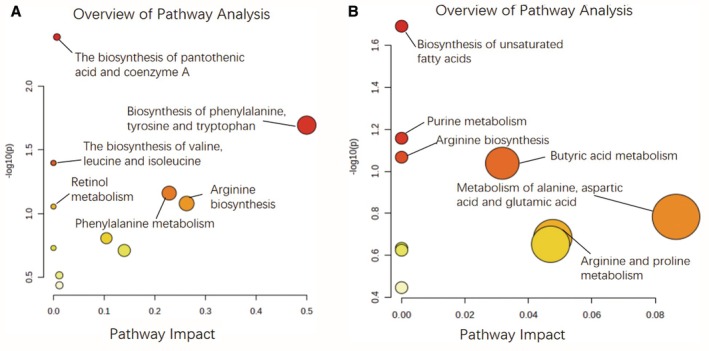
Metabolic pathway enrichment analysis of mouse serum. (A) ESI^+^ mode, (B) ESI^−^ mode.

Differential metabolites were analyzed using MetaboAnalyst 5.0 (http://www.metaboanalyst.ca) for metabolic pathway enrichment, with VIP scores plotted on the *x*‐axis and *p* values on the *y*‐axis. The results demonstrated that WP supplementation modulated multiple critical metabolic pathways in NAFLD mice, including arginine biosynthesis, unsaturated fatty acid biosynthesis, and tryptophan metabolism, as illustrated in Figure 9. These findings suggest WP exerts broad regulatory effects on key hepatic and systemic metabolic networks involved in NAFLD progression.

## 4 Discussion

While the therapeutic potential of whey protein (WP) in ameliorating hepatic lipid metabolism disorders is increasingly recognized (Jiang et al. 2024; Lee et al. 2021; Liu, Sun, et al. 2023; Liu, Wu, et al. 2023; Zumbro et al. 2021), the precise mechanistic pathways underlying its protective effects against NAFLD remain to be fully elucidated. Existing evidence highlights WP's capacity to reduce pro‐inflammatory cytokines and oxidative stress due to its rich bioactive peptides and cysteine content (Li, Paulsen, et al. 2022; Li, Zhu, et al. 2022; Yang et al. 2021). Building upon this, our study systematically demonstrates that WP intervention effectively attenuates HFD‐induced NAFLD progression not just through basic nutritional support, but by orchestrating a complex “bile acid‐gut microbiota‐liver” signaling network.

Consistent with previous clinical reports, our study revealed that dietary WP intervention markedly improved serum lipid profiles in NAFLD mice, specifically characterized by significant reductions in TC, TG, and LDL‐C, alongside a notable elevation in HDL‐C. This is consistent with prior clinical and experimental findings demonstrating that WP supplementation significantly reduces hepatic fat fractions and circulating triglycerides in patients with abdominal obesity (Sandby et al. 2024), and effectively improves glycemic responses while reducing NAFLD‐related biomarkers in metabolic syndrome models (Zumbro et al. 2021). Although the hypolipidemic mechanisms may involve multiple pathways, gut microbiota modulation appears particularly significant—as supported by recent studies indicating WP's capacity to restore microbial diversity and ameliorate intestinal permeability under high‐fat diet conditions (Boscaini et al. 2021; Gallo et al. 2024; Zou et al. 2023). These findings provide robust experimental support for developing WP‐based functional foods for NAFLD management.

WP administration significantly downregulated hepatic inflammatory markers (NLRP3, NF‐κB) and fibrogenic factors (CCL2, α‐SMA), while upregulating antioxidant defense systems (Nrf2, HO‐1) in NAFLD mice. Molecular docking analyses revealed stable interactions between β‐lactoglobulin (a major WP component) and pro‐inflammatory proteins (STAT3, RAGE, MMP9), suggesting direct anti‐inflammatory mechanisms. The gut‐liver axis represents another potential pathway, whereby WP enhances anti‐inflammatory short‐chain fatty acid (SCFA) production (Alhabeeb et al. 2021; Feng et al. 2022), activates GPR43‐mediated macrophage inhibition, and reduces TLR4‐dependent Kupffer cell activation through improved gut barrier function (Park et al. 2021; Wada et al. 2025; Yu et al. 2025). Although our current study did not directly measure intestinal barrier‐related indicators (such as tight junction proteins or intestinal permeability), the robust elevation of colonic SCFAs observed herein provides a strong theoretical basis for this hypothesized mechanism, which warrants further targeted investigations.

16S rRNA sequencing demonstrated significant enrichment of beneficial genera (*Lactobacillus*, *Ligilactobacillus*, *Paramuribaculum*, *Limosilactobacillus*) in WP‐treated mice. These bacterial taxa ferment dietary fibers into acetate, propionate, and butyrate while creating an acidic microenvironment that favors SCFA‐producing commensals (Liu et al. 2022; Portincasa et al. 2022; Vinelli et al. 2022). LC–MS/MS quantification confirmed elevated colonic SCFA levels, supporting the “gut microbiota‐SCFA‐liver axis” hypothesis (Wu et al. 2025).

Bile acid metabolism is intrinsically linked to NAFLD pathogenesis (Bing and Li 2022; Liu, Sun, et al. 2023; Liu, Wu, et al. 2023). Our findings indicate that WP significantly modulates serum bile acid profiles, notably reducing primary bile acids while elevating beneficial secondary conjugated forms, specifically taurodeoxycholic acid (TDCA) and tauroursodeoxycholic acid (TUDCA). This elevation highlights a critical gut‐liver crosstalk mechanism. Mechanistically, the initial transformation is likely driven by WP‐modulated gut microbiota, such as the promotion of specific microbial strains capable of epimerizing primary bile acids into secondary backbones (e.g., UDCA) via hydroxysteroid dehydrogenases, alongside the regulation of *Lactobacillus* (Hernández‐Gómez et al. 2021; Kang et al. 2025) and 7α‐dehydroxylating bacteria like *Clostridium scindens* (Foley et al. 2021; Lucas et al. 2025; Wise and Cummings 2022). Subsequently, these microbially‐derived secondary bile acids are reabsorbed via the enterohepatic circulation and undergo enhanced hepatic re‐conjugation with taurine. These integrated changes effectively restore hepatic CYP7A1/CYP27A1‐mediated bile acid synthesis and normalize FXR/SHP feedback regulation (Rizzolo et al. 2021; Shulpekova et al. 2022; Wang et al. 2025). Given that TUDCA is a well‐documented hepatoprotective agent that alleviates endoplasmic reticulum stress, this gut‐microbiota‐driven and hepatic‐conjugated bile acid transformation collectively reduces hepatotoxic accumulation, representing a novel therapeutic mechanism of WP in NAFLD (Li et al. 2024; Wang et al. 2024).

Serum metabolomics revealed WP‐mediated upregulation of EPA, LPC 20:5, and other lipid mediators that enhance fatty acid oxidation and anti‐inflammatory responses. Concurrent elevation of (±)19(20)‐DiHDPA and 11‐keto testosterone suggests improved antioxidant capacity and lipolysis, while estrone and nicotianamine optimization indicates better glucoregulatory function (Fabjanowska et al. 2023; Paulukinas and Penning 2023). These metabolic improvements synergize with WP's microbiota/bile acid modulatory effects to combat hepatic steatosis, inflammation, and fibrogenesis.

## 5 Conclusion

This study systematically elucidates WP's multi‐target mechanisms against NAFLD: (1) enhancing cholesterol catabolism via CYP7A1/CYP27A1 while optimizing bile acid composition; (2) enriching *Lactobacillus* to boost SCFA production, which is widely recognized to support gut barrier maintenance; (3) suppressing NLRP3/NF‐κB inflammation and activating Nrf2/HO‐1 antioxidant pathways; (4) direct anti‐inflammatory effects through β‐lactoglobulin‐STAT3/RAGE/MMP9 interactions. The integrated “bile acid‐gut microbiota‐liver signaling” network provides a theoretical foundation for WP's clinical application in NAFLD management.

## Author Contributions

**Dongjin Xu:** conceptualization (equal), writing – original draft preparation (equal). **Biru Qiu:** conceptualization (equal), methodology (equal), writing – original draft preparation (equal). **Xueyun Dong:** methodology (equal), visualization (equal). **Jiajun Tan:** software. **Yunhan Xie:** resources (equal). **Yuanyuan Wan:** resources (equal). **Chenghai Chu:** resources (equal). **Chunrun Miao:** resources (equal). **Asmaa Ali:** formal analysis (equal), writing – review and editing (equal), supervision (equal). **Min Chen:** validation (equal). **Jiayuan He:** investigation (equal), validation (equal). **Liang Wu:** investigation (equal), writing – review and editing (equal), supervision (equal), funding acquisition. **Jiayong Xie:** writing – review and editing (equal), visualization (equal).

## Funding

This work was supported by Zhenjiang Science and Technology Innovation Fund (Key R&D Program‐Social Development) Project, SH2023073. Taizhou Science and Technology Support Program (Social Development) Project, SSF20230126.

## Ethics Statement

All animal experiments were carried out with approval by the Ethics Committee of Jiangsu University (protocol code UJS‐IACUC‐AP‐2022032011 and date of approval: January 2022) and adhered to the 3R principles.

## Conflicts of Interest

The authors declare no conflicts of interest.

## Supporting information


**Data S1:** fsn371655‐sup‐0001‐Supinfo.docx.

## Data Availability

The data presented in this study are available on request from the corresponding authors.
