# Supplementary material for: Multi‐Target Mechanisms of Whey Protein Against NAFLD: Integrating Bile Acid Metabolism, Gut Microbiota and Hepatic Inflammation
Source: Food Sci Nutr. 2026 Mar 16;14(3):e71655. doi: 10.1002/fsn3.71655 (PMC13093742; doi:10.1002/fsn3.71655)
Supplement: Supplementary file 1 — Data S1: fsn371655‐sup‐0001‐Supinfo.docx. [file FSN3-14-e71655-s001.docx]

**Supplementary Information**

**1. Nutritional Composition of High-Fat Diet (HFD) Versus Regular Chow Diet**

| **Group** **and** **Component** | HFD | HFD+WP | Regular Diet |
| --- | --- | --- | --- |
| Casein (g) | 200 | 0 | 200 |
| Whey protein (g) | 0 | 189 | 0 |
| L-Cystine (g) | 3 | 3 | 3 |
| Corn Starch (g) | 0 | 0 | 315 |
| Maltodextrin (g) | 125 | 125 | 35 |
| Cellulose (g) | 50 | 50 | 50 |
| Soybean Oil (g) | 25 | 25 | 25 |
| Lard (g) | 245 | 245 | 20 |
| Mineral Mix (g) | 10 | 10 | 10 |
| DiCalcium Phosphate (g) | 13 | 13 | 13 |
| Calcium Carbonate (g) | 5.5 | 5.5 | 5.5 |
| Potassium Citrate, 1H₂O (g) | 16.5 | 16.5 | 16.5 |
| Vitamin Mix (g) | 10 | 10 | 10 |
| Choline Bitartrate (g) | 2 | 2 | 2 |
| Energy(kJ/g) | 21.4 | 21.4 | 15.5 |
| Protein (kJ%) | 18 | 18 | 18 |
| Carbohydrate (kJ%) | 20 | 20 | 71 |
| Fat (kJ%) | 62 | 62 | 10 |

**2. Analysis of Serum Bile Acid Profile and Fecal Short-Chain Fatty Acids in Mice**

After centrifugation of mouse serum samples at 3000 rpm for 10 min, the supernatant was separated and stored at −80°C. Prior to analysis, 100 μL of serum was mixed with 200 μL of ice-cold methanol (containing 0.1% formic acid), vortexed for 30 s, and centrifuged at 12,000 rpm at 4°C for 10 min. The supernatant was then filtered through a 0.22 μm membrane for analysis. Serum bile acids were analyzed using an Agilent 1290/6460 LC-MS/MS system equipped with a ZORBAX Eclipse Plus C18 column (2.1 × 100 mm, 1.7 μm). The mobile phases consisted of (A) 0.1% formic acid in water and (B) 0.1% formic acid in acetonitrile, with a gradient elution program as follows: 0–2 min (10% B), 2–4 min (10%→50% B), 4–6 min (50%→80% B), 6–8 min (80% B), and 8–10 min (10% B). Mass spectrometry detection was performed in negative electrospray ionization (ESI) mode using multiple reaction monitoring (MRM) for bile acid quantification.

For fecal short-chain fatty acid (SCFA) analysis, freeze-dried fecal samples (50 mg) were weighed, homogenized in 1 mL of 50% methanol (containing 4-methylvaleric acid as an internal standard), vortexed for 1 min, and sonicated for 20 min. After centrifugation at 12,000 rpm for 10 min, the supernatant was filtered through a 0.22 μm organic-phase membrane. For GC-MS analysis, 100 μL of the supernatant was derivatized with 50 μL of 25% N-methyl-N-(trimethylsilyl)trifluoroacetamide (MSTFA) at 70°C for 30 min prior to injection. SCFAs were analyzed using an Agilent 7890B/5977B GC-MS system with a DB-FFAP column (30 m × 0.25 mm, 0.25 μm). The temperature gradient was set as follows: initial hold at 60°C for 1 min, ramped to 200°C at 10°C/min, then to 250°C at 5°C/min, and held for 5 min. Detection was performed using electron impact ionization (EI) in selected ion monitoring (SIM) mode.

3. Table S1 qPCR Primers sequences

| Gene | Upstream primer (5′ → 3′) | Downstream primer(5′ → 3′) |
| --- | --- | --- |
| Nrf2 | CAGTGCTCCTATGCGTGAAT | TGGGCAACCTGGGAGTAGTA |
| HO-1 | AAGCCGAGAATGCTGAGTTCA | GCCGTGTAGATATGGTACAAGGA |
| GPX1 | GCAACCGCTTCTGGAAACAG | TCTTGGCGTTCTCCTGATGC |
| CAT | TGGGATCTCTTCAGGGAACA | GGCGAAGACGACCAAGATG |
| NLRP3 | GATCTTCGCTGCGATCAACAG | CGTGCATTATCTGAACCCCAC |
| Caspase-1 | GAGCTGGACTGCGTGTGAAT | TGGGCACAGACAGTGTTGTTG |
| IL-1β | GCAACTGTTCCTGAACTCAACT | ATCTTTTGGGGTCCGTCAACT |
| TNF-α | CCCTCACACTCAGATCATCTTCT | GCTACGACGTGGGCTACAG |
| NF-κB | CCTGGAGCAAGAGCAAACATC | AGAGCTGATCCGAGATGTTGTCA |
| CCL2 | TTAAAAACCTGGATCGGAACCAA | GCATTAGCTTCAGATTTACGGGT |
| α-SMA | CTGACAGAGGCACCACTGAA | CATCTCCAGAGTCCAGCACAAT |
| CYP7A1 | CTGCAACTCCACAAGATCACCA | CAGGGATTTCTCTGTGGCTGT |
| CYP27A1 | TGCTGGGATCTGTGTCCTTG | AGCATCCAGGTAGCCAGACC |
| FXR | CCTCCACCTACACCACCAAC | GCTGGGTGATGATGGTGATG |
| SHP | TGTGGACGCCTACCTGAATC | TCCACTGTGGCTTTGTCCTTC |
| GAPDH | AGGTCGGTGTGAACGGATTTG | TGTAGACCATGTAGTTGAGGTCA |

**4. Table S2. Serum metabolic alterations induced by WP in NAFLD mice (ESI+)**

| No | Metabolites | Formula | HMDB | m/z | RT [min] | which-  max |
| --- | --- | --- | --- | --- | --- | --- |
| 1 | L-(-)-Methionine | C5H11NO2S | HMDB0000696 | 150.05812 | 1.877 | ↓ |
| 2 | Propionylcarnitine | C10H19NO4 | HMDB0000824 | 218.13833 | 2.861 | ↓ |
| 3 | L-Tyrosine | C9H11NO3 | HMDB0000158 | 182.08097 | 1.63 | ↓ |
| 4 | Pantothenic acid | C9H17NO5 | HMDB0000210 | 220.11758 | 4.963 | ↓ |
| 5 | Valine | C5H11NO2 | HMDB0000883 | 118.0861 | 1.462 | ↓ |
| 6 | L-Valine | C5H11NO2 | HMDB0000883 | 118.08612 | 1.698 | ↓ |
| 7 | 3-(3,4-dihydroxyphenyl)propanoic acid | C9H10O4 | HMDB0000423 | 165.05442 | 1.66 | ↓ |
| 8 | Hexadecanamide | C16H33NO | HMDB0012273 | 256.2628 | 10.152 | ↑ |
| 9 | Stearamide | C18H37NO | HMDB0034146 | 284.29386 | 10.241 | ↑ |
| 10 | Ecgonine methyl ester | C10H17NO3 | HMDB0006406 | 200.12773 | 5.361 | ↓ |
| 11 | 2,6-Dihydroxypurine | C5H4N4O2 | HMDB0000292 | 153.04052 | 4.611 | ↓ |
| 12 | Stearoyl Ethanolamide | C20H41NO2 | HMDB0013078 | 328.31998 | 9.697 | ↑ |
| 13 | Ouabain | C29H44O12 | HMDB0015224 | 567.27357 | 8.137 | ↑ |
| 14 | L-Citrulline | C6H13N3O3 | HMDB0000904 | 176.10329 | 11.612 | ↑ |
| 15 | Norbuprenorphine | C25H35NO4 | HMDB0060546 | 414.26871 | 8.414 | ↑ |
| 16 | 1-Phenyl-3-methyl-5-pyrazolone | C10H10N2O | HMDB0251697 | 175.08632 | 4.849 | ↓ |
| 17 | 3-Chloro-L-tyrosine | C9H10ClNO3 | HMDB0001885 | 233.06926 | 2.401 | ↑ |
| 18 | Androsterone | C19H30O2 | HMDB0000031 | 291.22717 | 8.334 | ↑ |
| 19 | gamma-Glutamylleucine | C11H20N2O5 | HMDB0011171 | 261.14372 | 5.302 | ↓ |
| 20 | Vitamin A | C20H30O | HMDB0000305 | 269.22562 | 9.459 | ↑ |
| 21 | (-)-secoisolariciresinol | C20H26O6 | HMDB0013692 | 363.18169 | 10.016 | ↑ |
| 22 | Muramic acid | C9H17NO7 | HMDB0003254 | 252.10728 | 1.323 | ↓ |
| 23 | Isoeugenol | C10H12O2 | HMDB0005802 | 165.09113 | 5.573 | ↓ |

**5. Table S3. Serum metabolic alterations induced by WP in NAFLD mice (ESI-)**

| NO | Metabolite | Formula | HMDB | m/z | RT [min] | which-max |
| --- | --- | --- | --- | --- | --- | --- |
| 1 | Uric acid | C5H4N4O3 | HMDB0000289 | 167.02038 | 1.923 | ↓ |
| 2 | Xanthosine | C10H12N4O6 | HMDB0000299 | 283.06754 | 4.612 | ↓ |
| 3 | 5-Methoxyindoleacetic acid | C11H11NO3 | HMDB0004096 | 204.06591 | 5.647 | ↑ |
| 4 | p-Anisaldehyde | C8H8O2 | HMDB0029686 | 181.04994 | 5.134 | ↓ |
| 5 | Arachidic acid | C20H40O2 | HMDB0002212 | 311.29451 | 11.764 | ↑ |
| 6 | 13Z,16Z-Docosadienoic Acid | C22H40O2 | HMDB0061714 | 335.29447 | 11.285 | ↑ |
| 7 | Trichloroacetic acid | C2HCl3O2 | HMDB0042048 | 160.89641 | 5.127 | ↑ |
| 8 | N2-Acetyl-L-ornithine | C7H14N2O3 | HMDB0003357 | 173.09246 | 1.412 | ↑ |
| 9 | Ginkgolic Acid | C22H34O3 | HMDB0033897 | 327.23186 | 8.856 | ↑ |
| 10 | Triethyl citrate | C12H20O7 | HMDB0034263 | 275.11283 | 4.99 | ↓ |
| 11 | 4-Aminobutyric acid | C4H9NO2 | HMDB0000112 | 102.05591 | 1.361 | ↓ |
| 12 | L-Thyroxine | C15H1I4NO4 | HMDB0000248 | 775.67869 | 6.241 | ↑ |
| 13 | Pterostilbene | C16H16O3 | HMDB0303379 | 255.10203 | 6.146 | ↓ |
| 14 | Orotic acid | C5H4N2O4 | HMDB0000226 | 155.00948 | 1.727 | ↑ |
| 15 | Bergenin | C14H16O9 | HMDB0249087 | 327.07127 | 5.227 | ↑ |
| 16 | Artesunate | C19H28O8 | HMDB0240267 | 383.16998 | 5.628 | ↑ |
| 17 | 4-Chlorophenol | C6H5ClO | HMDB0246398 | 126.99533 | 7.05 | ↑ |
| 18 | Ginkgolide B | C20H24O10 | HMDB0036861 | 423.12823 | 5.947 | ↑ |
| 19 | Phloretin | C15H14O5 | HMDB0003306 | 273.07629 | 5.604 | ↑ |
| 20 | Schisandrin A | C24H32O6 | HMDB0251013 | 415.21087 | 7.199 | ↑ |
| 21 | Docosapentaenoic acid | C22H34O2 | HMDB0246621 | 329.24717 | 9.857 | ↑ |
| 22 | Eicosapentaenoic acid | C20H30O2 | HMDB0001999 | 301.21589 | 9.176 | ↑ |
| 23 | Apo-13-zeaxanthinone | C18H26O2 | HMDB0302026 | 273.18507 | 8.435 | ↑ |
